# A Quarter Century History of *ATML1* Gene Research

**DOI:** 10.3390/plants10020290

**Published:** 2021-02-03

**Authors:** Hiroyuki Iida, Shinobu Takada

**Affiliations:** 1 Institute of Biotechnology, HiLIFE, University of Helsinki, Viikinkaari 1, 00014 Helsinki, Finland; hiroyuki.iida@helsinki.fi; 2 Department of Biological Sciences, Graduate School of Science, Osaka University, 1–1 Machikaneyama, Toyonaka, Osaka 560-0043, Japan

**Keywords:** embryogenesis, epidermal development, *ATML1*, transcriptional regulation, post-transcriptional regulation

## Abstract

The cloning of the *ATML1* gene, encoding an HD-ZIP class IV transcription factor, was first reported in 1996. Because *ATML1* mRNA was preferentially detected in the shoot epidermis, cis-regulatory sequences of *ATML1* have been used to drive gene expression in the outermost cells of the shoot apical meristem and leaves, even before the function of *ATML1* was understood. Later studies revealed that *ATML1* is required for developmental processes related to shoot epidermal specification and differentiation. Consistent with its central role in epidermal development, *ATML1* activity has been revealed to be restricted to the outermost cells via several regulatory mechanisms. In this review, we look back on the history of *ATML1* research and provide a perspective for future studies.

## 1. Epidermis Formation and Its Role in Plant Development

*ATML1* was first reported ~25 years ago as a gene that is expressed in the epidermis of *Arabidopsis thaliana*. The epidermis is the outermost cell layer of land plants, first formed during early embryogenesis. In *Arabidopsis thaliana*, embryo proper cells during the eight-cell stage undergo tangential cell divisions, generating outer and inner daughter cells. The outer daughter cells exhibit mostly anticlinal cell divisions to help maintain the single cell layer and are eventually differentiated into the epidermal cells.

The epidermis is positioned at the interface between plants and the environment; therefore, specialized cell types and structures in the epidermis facilitate abiotic and biotic stress responses. For example, stomatal guard cells and trichomes enable efficient gas exchange and prevent insect herbivory, respectively [1,2]. Additionally, the cuticle, a hydrophobic lipid layer, is deposited on the outermost surface of the shoot epidermal cell walls, in order to prevent water loss due to the dry terrestrial environment [3].

In addition to its protective functions, the epidermis plays an important role in controlling plant growth. Brassinosteroid (BR) is a plant hormone promoting cell division and expansion and the BR response and biosynthetic mutants show a severe dwarf phenotype. Activation of BR signaling in the outermost layer of those mutants rescued their dwarf phenotypes, whereas inactivation of BR signaling in the wild-type epidermal layer made plants small. These observations suggest that the epidermal layer restricts the extent of shoot growth depending on the activity of BR signaling [4]. It has also been reported that production of very-long-chain fatty acids (VLCFAs) in the epidermis, which are components of the cuticle wax, is required for repressing cell proliferation in the vascular tissue [5,6]. Observed increase in cytokinin, a plant hormone that promotes cell proliferation, in VLCFA-deficient seedlings suggests that VLCFAs or their derivatives play a role in repressing cytokinin biosynthesis in the vasculature [5].

The epidermis also influences pattern formation in the shoot apical meristem (SAM), as several reports have suggested that certain microRNAs (miRNAs), generated in the outermost cell layer of the SAM, form an inhibitory gradient that contributes to the positioning of the stem cell niche at away from the epidermis [7,8]. Consistently, epidermal deficient mutants often show ectopic SAM activity in the leaves [9].

**Figure 1:**
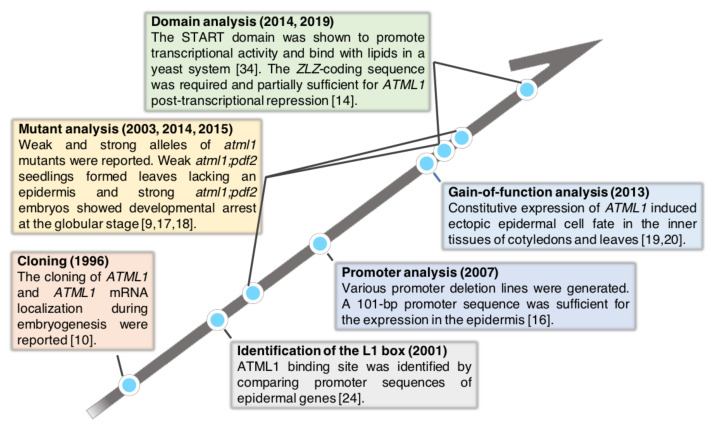
Timeline of the *ATML1* research. The history of *ATML1* gene research is summarized. Each box shows important findings in the indicated year.

Molecular genetic analysis has revealed many genes that are required for epidermal cell differentiation. Among those genes, *ARABIDOPSIS THALIANA MERISTEM L1 LAYER* (*ATML1*), which encodes an HD-ZIP class IV transcription factor, has been shown to be a master regulator for shoot epidermal cell identity in *Arabidopsis thaliana* (Figure 1). This review summarizes *ATML1* research of the last 25 years and provides a future perspective in the field.

## 2. Cloning of the *ATML1* Gene and Analysis of Its Expression Pattern

**Figure 2:**
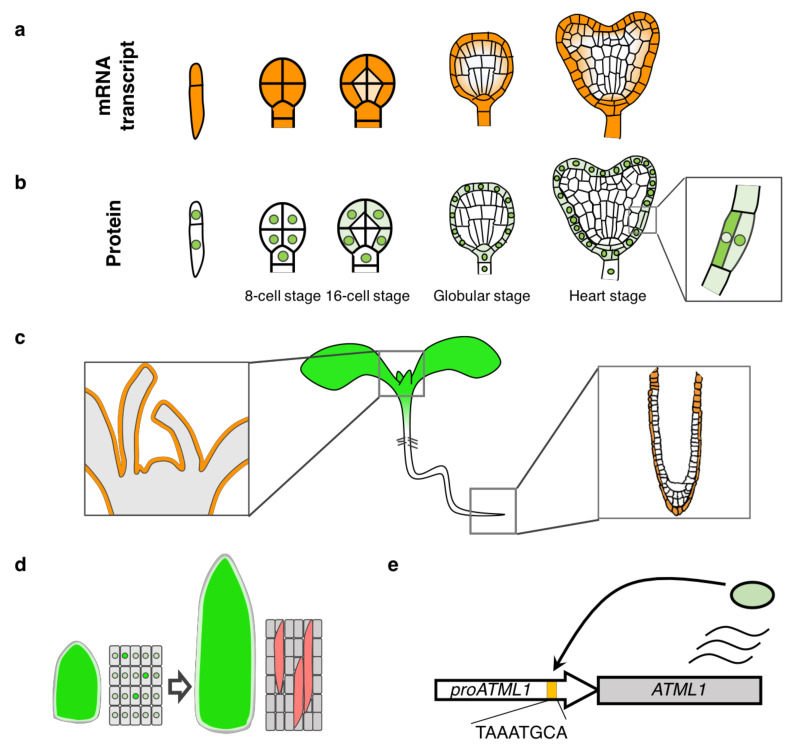
Schematic of *ATML1* transcript and ATML1 protein localization. (**a**) *ATML1* is transcribed strongly in the outermost cells and weakly in the inner cells of the embryos (**b**) ATML1 protein accumulation is observed only in the surface cells from the 16-cell stage. ATML1 protein is exclusively localized in the nuclei until the 8-cell stage. From the 16-cell stage onwards, ATML1 protein is present in both nuclei and cytosol of the outermost cells of the embryo-proper, while it only remains to accumulate in the nuclei of the suspensor cells. When the epidermal cells undergo aberrant periclinal divisions, ATML1 nuclear accumulation becomes weaker in the inner daughter cells compared with the outer daughter cells. (**c**) *ATML1* activity during post-embryonic development in the shoot apex (left) and the root tip (right). (**d**) When accumulated above the threshold level during the G2 phase, ATML1 promotes giant cell formation in the sepal epidermis. (**e**) Feedback regulation of *ATML1* via the L1 box in the promoter (*proATML1*). Orange: *ATML1* transcription, green in the cell: ATML1 protein, red: giant cells, yellow box in the *ATML1* promoter: an L1 box.

*ATML1* was first identified in a screen searching for cDNA clones that have a sequence homology with an ovule-enriched homeobox gene, O39 in orchid, by plaque hybridization [10,11]. ‘*ATML1′* was initially named as an acronym of *ARABIDOPSIS THALIANA MERISTEM L1 LAYER* (the outermost layer is called L1) although it is mistakenly written down as *AtML1* or has been described with the acronym of *ARABIDOPSIS THALIANA MERISTEM LAYER1*. *ATML1* belongs to the HD-ZIP class IV family transcription factors, including 16 members in *Arabidopsis thaliana*. The first identified member of the HD-ZIP class IV genes, *GLABRA2* (*GL2*), was involved in trichome and root hair patterning [12,13]. The first report showed that *ATML1* mRNA was detected specifically in the outermost cells of embryos, the SAM, and floral organs; *ATML1* is the first gene shown to be expressed in a specific cell layer of plant tissues [10] (Figure 2a,c).

In the first report, in situ hybridization experiments showed preferential accumulation of *ATML1* mRNA in the outermost cells of the SAM, leaf primordia, and floral organs. During embryogenesis, *ATML1* mRNA was detected in the apical cell of the one-cell stage embryo and its localization was restricted to the outermost cells of the embryo-proper from the early globular stage onwards [10].

After the first report, *ATML1* expression pattern has been re-examined in more details by several researchers [14,15,16]. *ATML1* promoter activity was also detected in the outermost cells of the root meristem [15] (Figure 2c). Detailed expression analysis in the embryos, with the aid of a sensitive reporter gene, revealed that *ATML1* promoter was also active in the basal cell of the one-cell stage embryo and in the suspensor cells (Figure 2a). Furthermore, when the reporter was fused with a destruction-box motif, which degrades the reporter protein at anaphase and enables detection of only newly synthesized protein after cell division, *ATML1* promoter activity was hardly detected in the inner cells of the 16-cell stage embryos, suggesting that *ATML1* transcription is restricted to the outermost cells from the 16-cell stage [16]. Recently, we reported that although *ATML1* was strongly expressed in the outermost cells, weak expression was also detected in the inner cells of the embryos, especially in the subepidermal cells [14] (Figure 2a).

In situ localization of *ATML1* transcripts reported in 1996 implied its role in epidermis development, which was later proved in 2003.

## 3. *ATML1* Functions in Plant Development

*ATML1* loss-of-function mutant phenotypes were first described in 2003 and its role in plant development was explored. The *atml1-1* single mutant, which has a T-DNA insertion near the 3′ end of the ORF, did not show a clear abnormality [17]. *PROTODERMAL FACTOR2* (*PDF2*), an *ATML1* paralog expressed in the epidermis, functions redundantly with *ATML1*; the *atml1-1;pdf2-1* double mutant is seedling-lethal harboring reduced cotyledons and narrow leaves lacking an epidermis [17]. Transcript levels of some epidermal genes were reduced in the *atml1-1;pdf2-1* mutant background, suggesting that *ATML1* and *PDF2* are required for epidermal differentiation. After 11 years, *atml1-1* was shown to be a weak allele mutant and a new *ATML1* null mutation, caused by a T-DNA insertion in the homeobox, combined with a *PDF2* mutation turned out to cause developmental arrest at the globular stage. The outermost cells of the arrested *atml1;pdf2* embryos were swollen and often underwent unusual periclinal cell divisions, suggesting that epidermal cell identity was lost in those embryos [9,18]. These results revealed that *ATML1* and *PDF2* are required for proper embryo development as well as epidermal specification. Considering that the epidermis is the first tissue formed during embryogenesis, the epidermis formation might be a prerequisite for the proper progression of embryogenesis.

Gain-of-function experiments have also been performed to assess the ability of *ATML1* to promote epidermal cell differentiation. When *ATML1* expression was constitutively induced in whole seedlings, epidermal traits such as stomatal guard cells and trichomes were found ectopically in the inner tissues of cotyledons or leaves [19,20]. Given that ectopic expression of a guard-cell marker was detected in *ATML1*-overexpressing roots, ATML1 should promote shoot but not root epidermal cell identity [20]. Furthermore, expression of *ATML1* and *PDF2* fused to a transcriptional repressor sequence (called EAR or SRDX motif) caused organ fusions and high permeability to a hydrophobic dye, which are typical phenotypes related to cuticle formation defects [9,21]. Expression of *ATML1-SRDX*, which is supposed to downregulate target genes of ATML1, caused de-differentiation of the epidermis even at late stages of seedling development, suggesting that ATML1 and/or its target genes are required for epidermal cell fate maintenance [21]. Additionally, *ATML1* promotes the differentiation of giant cells during flower development; in *Arabidopsis thaliana*, sepal epidermal cells come in different sizes: large ‘giant cells’ and small cells. The *atml1-2* mutation lacked giant cell formation, while *ATML1* overexpression resulted in sepals consisting of mostly giant cells [22,23]. Fluctuations of ATML1 protein levels occurred in sepal epidermal cells and when its level was above a certain threshold during the G2 phase, these cells would undergo endoreduplication and became giant cells [22] (Figure 2d). Therefore, *ATML1* is a key regulator not only for epidermal specification during embryogenesis but also for maintaining and specializing epidermal cells even at later development stages.

## 4. Transcriptional Regulation of the *ATML1* Gene

Multiple studies address how *ATML1* transcription is preferentially activated in the outermost cells, for example, by analyzing cis-regulatory sequences of *ATML1* [15,16,24]. Sequence comparison of L1-specific promoters, including *ATML1*, has identified a conserved cis-regulatory sequence, called L1 box. Direct binding of ATML1 and PDF2 to the L1 box has been shown in vitro and mutations in the L1 box abolished the expression of an epidermal gene, *PDF1* [17,24]. This has provided a molecular basis for epidermal gene activation by ATML1: *ATML1* activates epidermis-specific gene expression through its direct binding to the L1 box. Consistently, expression of epidermal genes that contain the L1 box in their regulatory regions was increased and decreased in *ATML1*-overexpressing and *atml1;pdf2* mutant plants, respectively [20]. Binding of ATML1 to the L1 box region was confirmed also in vivo by chromatin immunoprecipitation assays [18]. Because the *ATML1* promoter contains an L1 box sequence, it has been proposed that positive feedback regulation may be important for epidermis-specific expression of *ATML1* [24] (Figure 2e).

The whole genomic fragment of *ATML1* and the 3.4-kb promoter sequence, which has been widely used as a driver for epidermis-specific expression, drove the same expression pattern, suggesting that the 3.4-kb promoter sequence contains all the required cis-elements for proper *ATML1* expression [14,16]. For more precise understanding of *ATML1* transcriptional regulation, effects of deletions in the 3.4-kb *ATML1* promoter on the outermost-cell specific expression were examined in the embryos. Detailed promoter analysis has shown that a 101-bp *ATML1* promoter fragment, located upstream of the translational start site and contains an L1 box and a WUS-binding site, was sufficient for the expression in the outermost cells. Unexpectedly, even when mutating both the L1 box and WUS-binding site, reporter gene expression was reduced but still detected in the outermost cells, indicating that the expression in the epidermal cells can be achieved independently of these cis-elements. Consistently, *ATML1* expression was still detected in the outermost cells of *atml1-1;pdf2-1* embryos and leaves, suggesting that positive feedback regulation is not required for the outermost-cell specific promoter activity [16,20].

There are two scenarios that can explain the outermost cell-specific expression of *ATML1*: transcriptional activation in the outermost cells or transcriptional repression in the inner cells. Although it is difficult to distinguish these two possibilities, reporter gene expression was not activated in the inner cells of any promoter-deletion lines tested, implying that there is no transcriptional repressors that directly bind to the *ATML1* promoter and repress its expression in the inner cells of the embryos [16]. Therefore, only positive regulators appear to act on the *ATML1* promoter to activate expression in the outermost cells, suggesting that transcriptional activation in the outermost cells is more plausible. However, we cannot exclude the possibility that these positive regulators are repressed or inactivated in the inner cells of the embryos.

What could be the candidate factors that activate *ATML1* transcription in the outer cells? First, full epidermal cell identity seems not to be required for *ATML1* expression because *ATML1* promoter activity was still detected in the outermost mesophyll-looking cells in the *atml1-1;pdf2-1* mutant [20]. Secondly, *ATML1* expression in the epidermis was detected in mutants defective in auxin signaling, suggesting that auxin, a plant hormone known to direct many cell fate decisions, is not involved in the transcriptional control of *ATML1* [16]. Considering that cell fate decisions in plants largely depend on the position of the cell, *ATML1* expression could be influenced by the position of the cell; namely, the “surface” position [25]. In agreement with this idea, when the epidermal cells underwent aberrant periclinal divisions, only the outer daughter cells remained as epidermal cells while the inner daughter cells were differentiated into mesophyll cells [26]. In addition, *ATML1* was still transcribed in the outermost cells in a mutant defective in the control of cell division orientation (unpublished results from the authors). These results suggest that cell position but not cell lineage is required for *ATML1* transcription, as well for epidermal/mesophyll cell fate decisions. To date, positional cues required for epidermal/mesophyll cell fate changes have still remained unknown, however. Several mutants, in which *ATML1* transcription was decreased or lost, have been reported, implying that the causal genes of these mutants are positive regulators of *ATML1* transcription [27,28,29]. However, *ATML1* transcription could still be detected at early stages in these mutant embryos, suggesting that these causal genes may be involved, either directly or indirectly, in the maintenance of *ATML1* expression rather than its initiation [3]. Characterization of mutants that completely abolish or alter the *ATML1* expression pattern or direct purification of *ATML1* transcription-promoting molecules should be needed for further progress of *ATML1* research.

## 5. Post-transcriptional and Post-translational Regulation of *ATML1*

Several studies have implied post-transcriptional regulation of *ATML1*. In 2010, mutations in the *DICER-LIKE1* (*DCL1*) gene, which is required for correct processing of miRNAs, were shown to increase *ATML1* mRNA levels in suspensor cells whereas normally *ATML1* mRNA is under the detection limit in the wild-type suspensor cells [10,30]. This implies that *ATML1* mRNA is degraded in suspensor cells by unidentified miRNAs. However, the epidermis-specific *ATML1* mRNA accumulation was not affected in *dcl1* embryos, suggesting that miRNAs are not used as positional cues for epidermal specification [30]. Moreover, *ATML1* transcription and ATML1 protein accumulation were detected in both embryo proper and suspensor cells of wild-type embryos at similar levels using sensitive reporter lines, implying that protein accumulation is not affected despite the apparent differences in mRNA levels [14,16].

The idea of *ATML1* regulation at the protein level came from domain analysis. ATML1 protein is characterized by a homeodomain (HD), a zipper-loop-zipper (ZLZ) motif, a StAR-related lipid-transfer (START) domain and a START-associated domain (SAD) [31]. The HD domain is a DNA binding domain and ZLZ motif is required for dimerization [32]. The START domain is homologous to a lipid-sterol binding domain [33]. The function of SAD domain has not been elucidated. The START domains from ATML1, PDF2 and GL2 were implied to bind with lipids in a yeast system. Although transcriptional activity increased upon its binding with lipids, the START domain alone was not sufficient to confer transcriptional activation, suggesting that it influences the transcription in combination with other domains or co-factors [34]. Therefore, it is plausible that ATML1 is activated by interaction with signaling molecules including lipids through its START domain.

Recently, we have reported post-transcriptional regulation of *ATML1* in the embryos. We found that ATML1 protein was not detected in the inner cells of the embryos whereas *ATML1* was weakly transcribed in these cells (Figure 2a,b). In addition, when epidermal cells underwent periclinal cell divisions, nuclear localization of ATML1 was weaker in the inner daughter cells compared with the outer daughter cells (Figure 2b). Constitutive expression of *ATML1* also confirmed that nuclear localization was reduced in the inner tissues of the embryos. These observations suggest that ATML1 protein accumulation and nuclear localization are negatively regulated in the inner cells to restrict *ATML1* activity to the outermost cells. Treatment with a nuclear export inhibitor or domain deletions increased protein accumulation and nuclear localization of ATML1 in both the outermost and inner cells of the embryos, suggesting that *ATML1* activity was negatively regulated also in the outermost cells [14]. Because *ATML1* can potentially activate and maintain its own expression through positive feedback, post-transcriptional repression might be a reasonable mechanism to suppress excessive *ATML1* activity.

Domain deletion analysis revealed that the *ZLZ*-encoding sequence is required and partially sufficient for the post-transcriptional regulation. How the dimerization motif controls ATML1 protein accumulation and subcellular localization is unclear. One possibility is that protein-protein interaction is required for degradation or for nuclear export of ATML1 protein. The START domain was not necessary for the post-transcriptional repression in the outermost cells but was required for the repression of nuclear localization in the inner cells [14]. The START domain may, therefore, play a role in negatively regulating ATML1 activity in the inner cells by physical interaction with other molecules present in the inner tissues. To understand the mechanisms regulating *ATML1* activity at the post-transcriptional level, future work should focus on the identification of ATML1 interacting factors. DELLA proteins, which are central repressors of gibberellin (GA) signaling, were shown to interact with ATML1 and PDF2, probably to enable GA-dependent ATML1/PDF2 activation during seed germination [35]. In cotton, Gossypium barbadense Meristem Layer 1 (GbML1), an *ATML1* ortholog, has been shown to interact with a MYB domain transcription factor, GbMYB25 [32]. However, as far as we know, interaction of ATML1 or its orthologous protein with molecules that reduce nuclear accumulation or stability has not been reported yet.

## 6. Future Perspective

*ATML1* has become an indispensable tool and research target for elucidating epidermal differentiation. Despite being studied for more than two decades, several important questions remain to be answered. Firstly, molecular mechanisms that restrict *ATML1* activity in the outermost cells have not been identified. Molecules directly interacting with ATML1 protein or *ATML1* cis-regulatory sequences may convey “surface” positional cues. Secondly, although *ATML1* orthologues have been identified and shown to be preferentially expressed in the outermost cells in other plant species, loss-of-function mutant phenotypes and biological roles of those genes have yet to be examined [36,37,38]. From an evolutionary point of view, an *ATML1* ancestral gene should have acquired the function to promote the expression of genes required for epidermal features in the outermost cells during or before plant terrestrialization, as the epidermis is an essential tissue for land plants to live on dry lands. It would be interesting to identify the *ATML1* ancestral gene and study how its regulation has been evolved during the transition from aquatic to land plants. Further *ATML1* research would shed light on developmental and evolutionary processes of land plants.

## Data Availability

Not applicable.
